# Monocyte depletion attenuates the development of posttraumatic hydrocephalus and preserves white matter integrity after traumatic brain injury

**DOI:** 10.1371/journal.pone.0202722

**Published:** 2018-11-01

**Authors:** Hadijat M. Makinde, Talia B. Just, Carla M. Cuda, Nicola Bertolino, Daniele Procissi, Steven J. Schwulst

**Affiliations:** 1 Department of Surgery, Division of Trauma and Critical Care, Northwestern University, Chicago, Illinois, United States of America; 2 Department of Medicine, Division of Rheumatology, Northwestern University, Chicago, Illinois, United States of America; 3 Department of Radiology, Northwestern University, Chicago, Illinois, United States of America; 4 Department of Biomedical Engineering, Northwestern University, Chicago, Illinois, United States of America; Uniformed Services University, UNITED STATES

## Abstract

Monocytes are amongst the first cells recruited into the brain after traumatic brain injury (TBI). We have shown monocyte depletion 24 hours prior to TBI reduces brain edema, decreases neutrophil infiltration and improves behavioral outcomes. Additionally, both lesion and ventricle size correlate with poor neurologic outcome after TBI. Therefore, we aimed to determine the association between monocyte infiltration, lesion size, and ventricle volume. We hypothesized that monocyte depletion would attenuate lesion size, decrease ventricle enlargement, and preserve white matter in mice after TBI. C57BL/6 mice underwent pan monocyte depletion via intravenous injection of liposome-encapsulated clodronate. Control mice were injected with liposome-encapsulated PBS. TBI was induced via an open-head, controlled cortical impact. Mice were imaged using magnetic resonance imaging (MRI) at 1, 7, and 14 days post-injury to evaluate progression of lesion and to detect morphological changes associated with injury (3D T1-weighted MRI) including regional alterations in white matter patterns (multi-direction diffusion MRI). Lesion size and ventricle volume were measured using semi-automatic segmentation and active contour methods with the software program ITK-SNAP. Data was analyzed with the statistical software program PRISM. No significant effect of monocyte depletion on lesion size was detected using MRI following TBI (*p* = 0.4). However, progressive ventricle enlargement following TBI was observed to be attenuated in the monocyte-depleted cohort (5.3 ± 0.9mm^3^) as compared to the sham-depleted cohort (13.2 ± 3.1mm^3^; *p* = 0.02). Global white matter integrity and regional patterns were evaluated and quantified for each mouse after extracting fractional anisotropy maps from the multi-direction diffusion-MRI data using Siemens Syngo DTI analysis package. Fractional anisotropy (FA) values were preserved in the monocyte-depleted cohort (123.0 ± 4.4mm^3^) as compared to sham-depleted mice (94.9 ± 4.6mm^3^; *p* = 0.025) by 14 days post-TBI. All TBI mice exhibited FA values lower than those from a representative naïve control group with intact white matter tracts and FA~200 mm3). The MRI derived assessment of injury progression suggests that monocyte depletion at the time of injury may be a novel therapeutic strategy in the treatment of TBI. Furthermore, non-invasive longitudinal imaging allows for the evaluation of both TBI progression as well as therapeutic response over the course of injury.

## Introduction

The Centers for Disease Control and Injury Prevention estimates that over 2 million people sustain a traumatic brain injury (TBI) each year in the United States, contributing to over 30% of all injury related deaths [1, 2]. In fact, TBI related healthcare expenditures near 80 billion dollars annually with an average cost of 4 million dollars per person surviving a severe TBI [3–5]. The impact of TBI is highlighted not only by its high mortality rate but also by the significant long-term complications suffered by its survivors with the progressive development of motor, cognitive, and behavioral disorders [6–10]. The immune response to TBI plays a fundamental role in the development and progression of this subsequent neurodegeneration and represents a complex interplay between peripheral immunity and the resident immune system of the injured brain [11].

Data from our laboratory, as well as others, has demonstrated that monocytes comprise a larger percentage of the early inflammatory infiltrate within the injured brain than has been formerly known [12–14]. This lead us to hypothesize that infiltrating monocytes play a more significant role in the evolution of TBI than has been previously recognized. We recently published that infiltrating monocytes contribute to the development of cerebral edema, memory loss, and locomotor dysfunction after TBI [15]. Although these infiltrating cells are short-lived within the injury milieu, we demonstrated that targeted depletion of individual circulating monocytic subsets attenuates these neurocognitive and locomotor deficits. In general, behavioral assays are effective in evaluating the effects of TBI on neurocognitive function and could be used to evaluate novel therapeutic approaches. However, behavioral data generally only allows semi-quantitative evaluation of the long-term effects of TBI progression when damage is extensive and often irreversible. Histopathological evaluation provides a direct window into the cellular and tissue alterations associated with TBI at any stage of progression. However, these types of assays require tissue harvesting and are terminal thus not allowing for the longitudinal evaluation of TBI-induced neurodegenerative processes within the same subjects. For these reasons we sought to employ a rapid and noninvasive approach to assess TBI progression and therapeutic responses following monocyte depletion over the course of TBI. Recent literature has identified ventriculomegaly, also known as posttraumatic hydrocephalus, and its associated cortical thinning as a reliable imaging marker for the progression of TBI in rodent models [16, 17]. The incidence of clinically relevant posttraumatic hydrocephalus in brain-injured human patients has been estimated be as high as 45% and the development of posttraumatic hydrocephalus is tightly correlated with clinical outcomes [18–21]. In addition, advances in neuroimaging have allowed for in-vivo assessment of traumatic disruptions between neural connections. Threshold volumetric fractional anisotropy (FA), a measure of neural connectivity, can be used to longitudinally assess the integrity of white matter pathways and disruption of functional networks after TBI [22–24]. To the best of our knowledge no study to date has applied longitudinal diffusion-weighted and 3D high resolution morphological MRI to characterize the timing and progression of TBI in an immune-depleted model of TBI. Therefore, the aim of the current study is to use a reliable and rapid MRI longitudinal assay to test whether monocyte depletion therapy can ameliorate the effects of TBI by sparing affected brain tissue through the evaluation of 1) lesion and ventricle size and 2) white matter regional patterns associated with neuronal connectivity. We hypothesized that monocyte-depleted TBI mice will have greater preservation of injured tissue resulting in attenuation of ventricle enlargement as compared to sham-depleted TBI mice. We further hypothesized that this would result in preservation of white matter functional networks as manifest by smaller changes in FA pattern.

## Materials and methods

### Mice

C57BL/6 male mice (n = 15), 12–14 weeks old and 25g-30g, were bred and housed in a pathogen-free barrier facility within Northwestern University’s Center for Comparative Medicine. Animals were treated and cared for in accordance with the National Institutes of Health Guidelines for the Use of Laboratory Animals. The protocol was approved by the Northwestern University Institutional Animal Care and Use Committee (Protocol Number: IS00000554). All surgery was performed under ketamine and xylazine anesthesia, and post-operative analgesia was provided to minimize suffering. A control group, comprised of wildtype (WT) naïve mice, was used to provide a normal comparison to a set of brains not exhibiting any mechanical injury.

### Monocyte depletion

**Fig 1:**
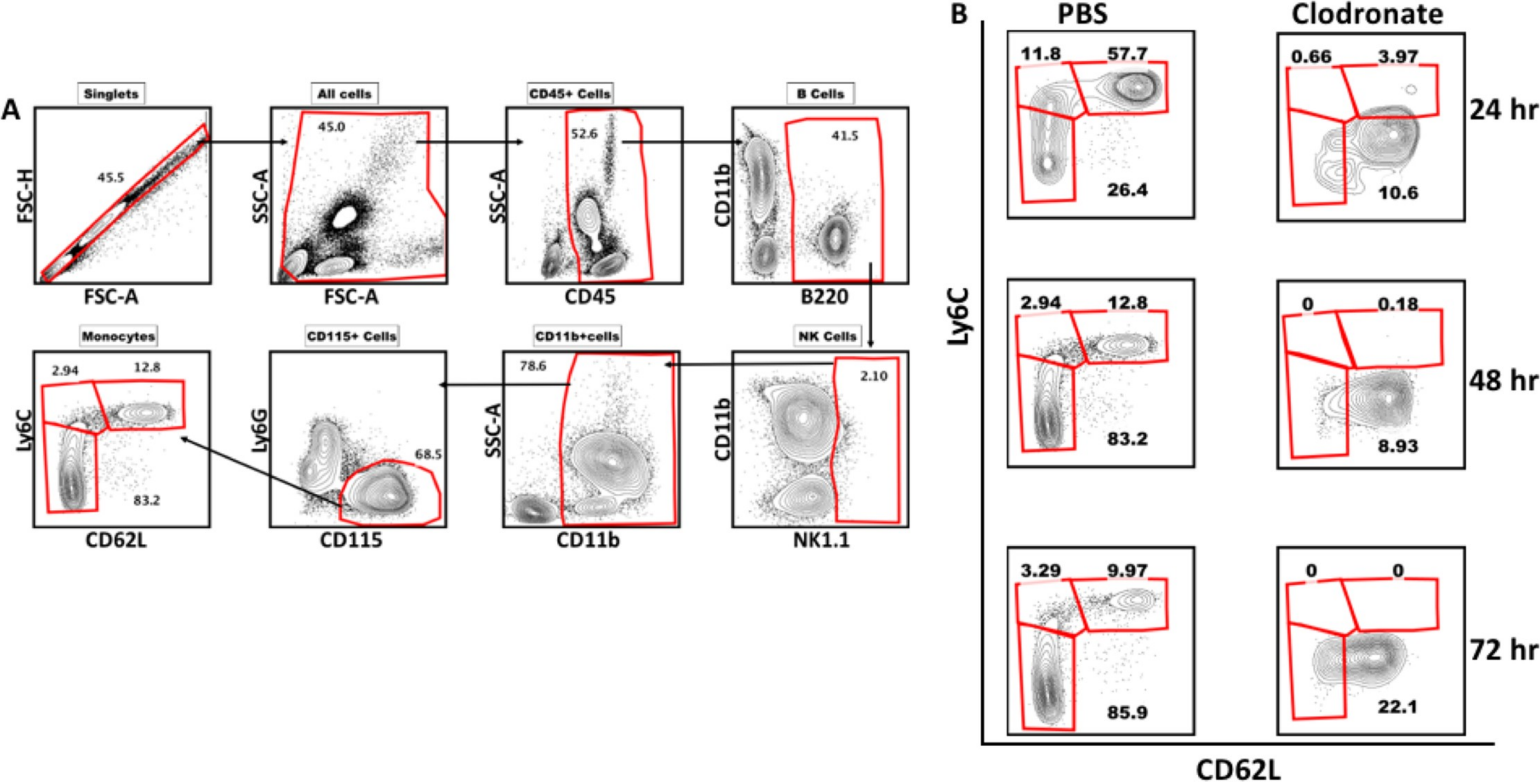
Confirmation of clodronate-induced monocyte depletion. **A)** Gating strategy for blood monocytes. **B)** Time line showing clodronate-induced monocyte depletion in mice from 24hrs to 72hrs after a single injection of clodronate as compared to sham-depleted (PBS) controls.

Mice underwent monocyte or sham depletion via intravenous injection of liposome encapsulated clodronate (Liposoma, Amsterdam) 24 hours prior to TBI as previously described [15]. Control mice were injected with liposome encapsulated PBS. Liposomes were administered according to the recommended dose of 10uL/1g of mouse. Each injection of liposome encapsulated clodronate abrogated circulating monocytes for 72 hours (**Fig 1**). Thereafter, monocytes return to normal circulating levels. Two additional doses of liposome encapsulated clodronate, or control liposomes, were administered at 72-hour intervals post injury.

### Controlled cortical impact

Controlled cortical impact was induced as previously described by our laboratory [15]. In brief, mice were anesthetized with 50 mg/kg Ketamine (Ketaset, Fort Dodge, IA) and 2.5 mg/kg Xylazine (Anased, Shenandoah, IA) via intraperitoneal injection. A 1cm scalp incision was performed and a 5mm craniectomy was performed 2 mm left of the sagittal suture and 2 mm rostral to the coronal suture. The dura was left intact. Mice were then placed in a stereotaxic operating frame and the impactor (Leica Biosystems Inc., Buffalo Grove, IL) was maneuvered into position. A controlled cortical impact was then applied with a 3mm impacting tip at a velocity of 2.5m/s and an impacting depth of 2mm with the dwell time set at 0.1s. Immediately following injury all animals had their scalps sealed with VetBond (3M) (Santa Cruz Animal Health, Dallas, TX). All animals received post injury analgesia with Buprenorphine SR (SR Veterinary Technologies, Windsor, CO) via subcutaneous injection and were allowed to recover in separate cages over a warming pad. Mice underwent imaging at 1, 7, and 14 days post TBI and were euthanized once imaging was complete.

### MRI acquisition and image processing methods

#### MRI acquisition

Each mouse was scanned using a 7T Clinscan MRI (Bruker, Billerica, MA) using a clinical grade software platform (Syngo, Siemens) at 1, 7 and 14 days post-injury. Twenty minutes prior to scanning each mouse was injected I.P. with a dose (~0.3 mmol/Kg) of MR gadolinium based contrast agent (Prohance, Bracco) with the aim of allowing enough time for systemic delivery to the brain and leakage of agent in those regions with a breached blood brain barrier (BBB). Twenty minutes after the injection of contrast each mouse was anesthetized using isoflurane mixed with 100% O_2_ and then placed in a dedicated holder with stereotactic alignment of the head. Respiration and body temperature were monitored with the dedicated physiological monitoring system (SAI, New Jersey, USA) throughout the imaging session with anesthesia continuously delivered through a nose cone. Using a four-channel mouse brain surface coil placed on the mouse head the following sequences were used to acquire the brain images: 1) a localizer tri-axial gradient echo sequence for positioning 2) a T1-weighted gradient echo 3D sequence with TR = and TE = and isotropic spatial resolution 150 micrometers 3) a multi-direction (64 directions) and multiple values diffusion gradient (0, 200, 800) 2D EPI sequence TR = 2000msec and TE = 25 msec with in plane resolution of 200 micrometers and 12 longitudinal slices covering the whole brain. Each scanning session lasted ~20–30 minutes.

#### MR image processing and region of interest selection criteria

Lesion size and ventricle volume were measured from the 3D brain images acquired at isotropic resolution of 150 micrometers using a combination of semi-automatic segmentation and active contour methods included in the freeware image processing software package ITK-SNAP http://www.itksnap.org/ [25]. The criteria for delineation of the different regions was based on the assumption that lesioned tissue (i.e. brain region affected by TBI) and ventricles exhibit a higher signal intensity as compared to the signal from normal surrounding brain tissue (~30% higher for lesion and ~30–50% higher for ventricle/CSF). The following steps (depicted schematically in Fig 1) were taken to ensure that the thresholded segmentation approach was reproducible across subjects and time-points:

1. Linear contrast adjustments were conducted on all the 3D MRI isotropic data sets at each time-point and for each cohort. To take into account inter-session variability we used the neck-muscle region as our reference tissue signal (muscle signal intensity (S.I.) ~ 80–90 (a.u.)) with the assumption that this does not change as a result of TBI progression.
2. A threshold window was chosen that facilitated visualization of hyperintense lesion tissue and ventricles (minimum S.I.~100 (a.u.) and maximum S.I. ~ 500 (a.u.)) in comparison to normal unaffected brain. This window was used to actively contour the regions of interest in all subjects. All voxels located spatially within the contour were classified as “lesion” and or “ventricle” even if the intensity fell outside the window. This was done under the assumption that there will be heterogeneity of signal within each region associated with sub-cellular and cellular events occurring simultaneously as injury progresses.
3. The active contouring was conducted by restricting the analysis to relevant brain region (i.e. avoiding anything outside the skull) and by taking into account known brain and ventricle structures. Two experienced preclinical imaging scientist (D.P. and N.B.) and a research scientist with experience and knowledge of brain anatomy (T.B.J.) oversaw the segmentation steps to ensure reproducibility across animals. Volume of the lesion and of the ventricular space for each mouse are expressed in mm^3^. Fractional anisotropy (FA) maps were generated using DTI processing packages contained in the image acquisition and processing platform (Syngo, Siemens) from the diffusion MRI images. In order to facilitate detection and quantification of FA differences across groups using the multi-slice data set, we used a similar threshold approach as the one described above. Using the 2D FA maps and the ROI tool contained in JIM 7.0 (Xinapse, Essex, UK), we segmented the area of high FA in each slice. Through this approach (described in more detail in [ISMRM abstract]) we generated whole brain 3D rendered FA surfaces and extracted the total volume for each subject. Using the assumption that reduction of FA reflects loss of white matter, we used the 3D volumetric data to compare across subjects and time-points to assess progression of neurodegeneration following TBI. The 3D FA rendered FA surfaces also enabled direct visualization of abnormal connectivity patterns reflected in morphometric alterations.

### Statistical methods

The data are reported as the mean ± standard error of the mean using the statistical software program Prism (GraphPad Software Inc., La Jolla, CA). Comparison between groups for lesion size ad ventricle size was performed with two-way repeated measures ANOVA with Bonferroni’s multiple comparison posttest. Comparison between groups for fractional anisotropy were performed with a Mann-Whitney U test.

## Results

### Segmentation of regions of interest

**Fig 2:**
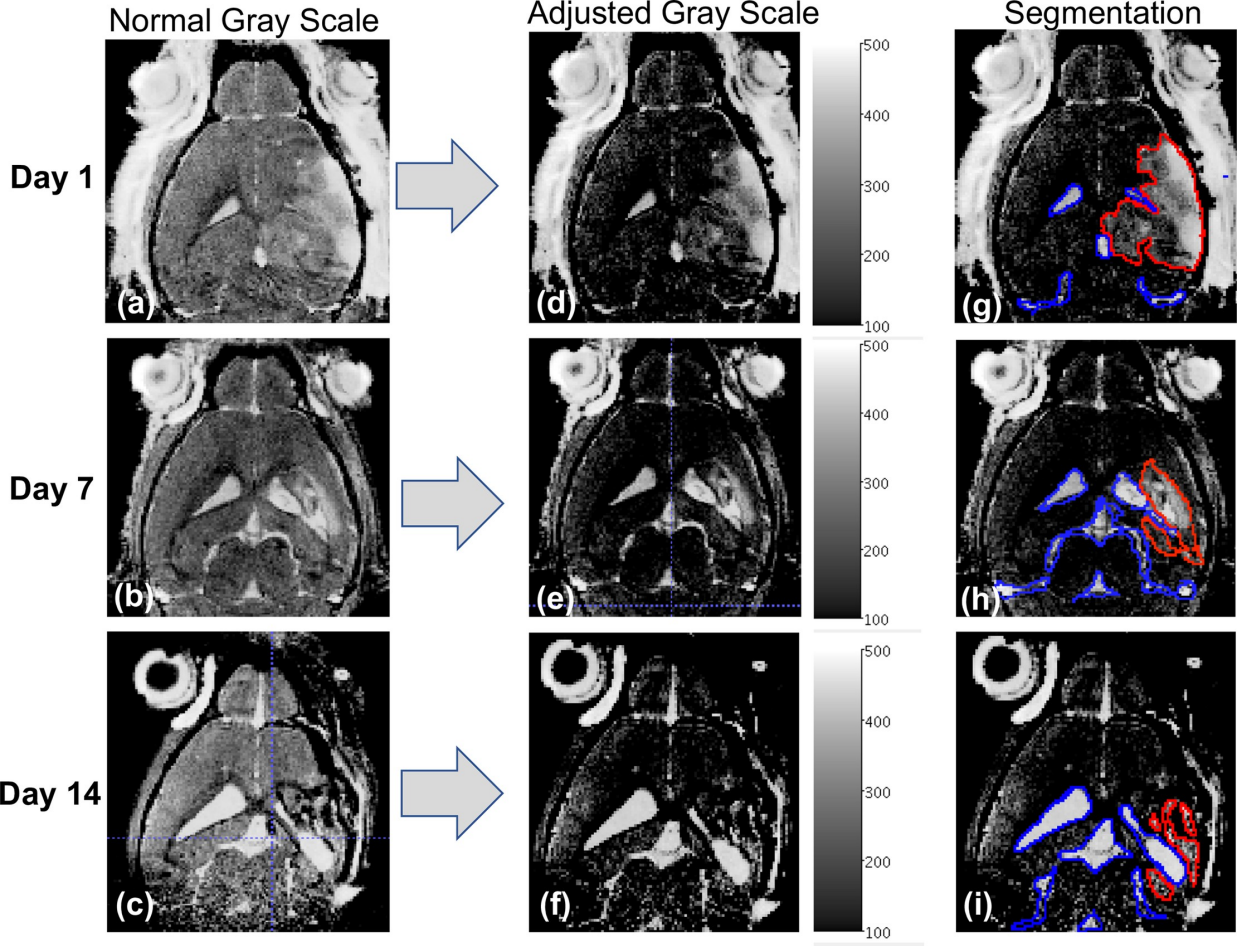
Schematic approach to define the threshold window for segmentation of ‘lesion area’ and the ventricular space. MR images corresponding to a TBI mouse at 1, 7, and 14 days post-TBI are shown. The schematic approach to define the threshold window for segmentation of ‘lesion area’ (RED outline) and the ventricular space (BLUE outline) is depicted with the first column showing the MR images at different time points in normal gray scale with contrast optimized for whole brain visualization. The center column depicts the corresponding images in an adjusted threshold scale (S.I.~100–500 a.u.) that facilitates visualization of both lesioned area and ventricular space. The last column superimposes the segmentation of regions of interest with RED outlining the lesion area and BLUE outlining the ventricular space. The progressive ventricular enlargement and corresponding reduction of the lesion area are clearly evident and are consistent with loss of brain tissue.

To more clearly illustrate our approach for segmentation of the relevant regions of interest as described in the method section, we present in **Fig 2** a schematic depiction of the steps taken to select and outline the relevant regions of interest for a representative mouse on day 1 (shortly after TBI), day 7, and day 14 post-TBI. One can note that selection of ventricle region as shown in blue (**Fig 2G–2I**) on days 1, 7, and 14, respectively, is straightforward and provides a robust measure of TBI progression. On the other hand, the delineation of the injury/lesioned region while straightforward on day 1 as shown in **Fig 2g** outlined in red, was challenging at 7 and 14 days after TBI when brain tissue loss/atrophy and ventricular enlargement become major features of TBI progression (**Fig 2H and 2I** outlined in blue).

### MRI is not able to detect the therapeutic effect of monocyte depletion on lesion size following TBI

**Fig 3:**
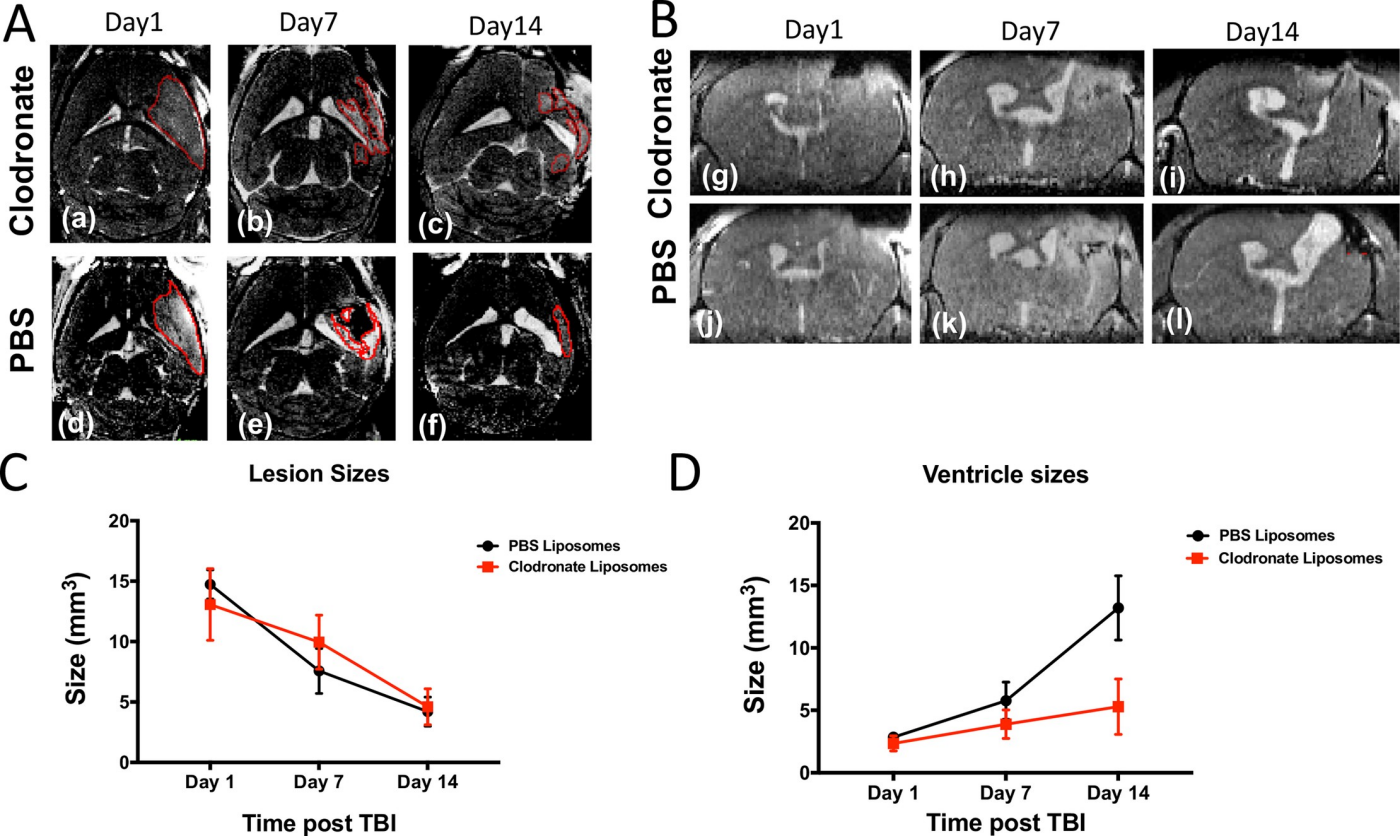
Clodronate-mediated monocyte depletion attenuates ventricle enlargement but not lesion size after traumatic brain injury. **A)** Representative axial T1W MR images of a TBI/Clodronate treated mouse (a-c) and a TBI/PBS treated mouse (d-f) on post-TBI days 1, 7, and 14. ‘Lesion area’ is outlined in RED. **B)** Representative coronal T1W MR images of the corresponding TBI/Clodronate treated mouse (g-i) and TBI/PBS treated mouse (j-l) at 1, 7, and 14 days post-TBI. The hyperattenuating area denotes the progressive enlargement of ventricle size over time. **C)** No significant difference in lesion size was detected at any time point between monocyte-depleted and sham-depleted mice (5 ± 1.1mm^3^ vs 7.3 ± 1.06mm^3^; *p* = 0.4). **D)** At 14 days post-TBI clodronate depletion markedly attenuated ventricle enlargement (5.3 ± 0.9mm^3^) as compared to sham-depleted mice(13.2 ± 3.1mm^3^; ** *p* = 0.02).

Prior studies have reported that depletion of inflammatory mediators such as neutrophils and IL-1β are able to reduce cerebral edema and prevent tissue loss after TBI [26, 27]. Our prior work has demonstrated that monocyte depletion also reduces cerebral edema and we hypothesized in the current study that monocyte depletion would reduce lesion size after TBI as well [15]. However, the prior depletion studies assessing lesion size after TBI have relied on post-mortem histopathology and gross calculation of hemispheric volume and did not focus on quantification of ventricle size. Since changes in lesion and ventricle size occur simultaneously, as brain tissue is lost and replaced by ventricular space, it is difficult to accurately characterize the size of a lesion by ex-vivo methods alone. With the use of 3D, contrast-enhanced, MRI we attempted to verify whether we could detect differences in lesion size progression between monocyte-depleted mice (n = 8) and sham-depleted mice (n = 7). No significant difference was observed between the two groups at any of the selected post-TBI time-points (1, 7 and 14 days) as shown **Fig 3**.

One should note that at day 7 we observed a lower, albeit not statistically significant, decrease in lesion size in the sham-depleted group as compared to the monocyte depleted group. This observed effect, together with the similar lesion size trends for the two cohorts, seems to challenge prior literature on immune depletion suggesting that monocyte depletion may not alter lesion size after TBI. However, as demonstrated in **Fig 2** we ascribe this to a limited ability in delineating and classifying regions of the brain as “lesion” in lieu of the dynamic and simultaneous interplay between lesion progression, loss of lesioned tissue, and progressive ventricle enlargement. We thus focused on MR derived assessment of ventricular space as a marker of TBI progression with the assumption that negative outcomes (i.e. increased loss of brain tissue) will be reflected by the increase in ventricle size.

### Monocyte depletion attenuates ventricle enlargement after TBI

**Fig 4:**
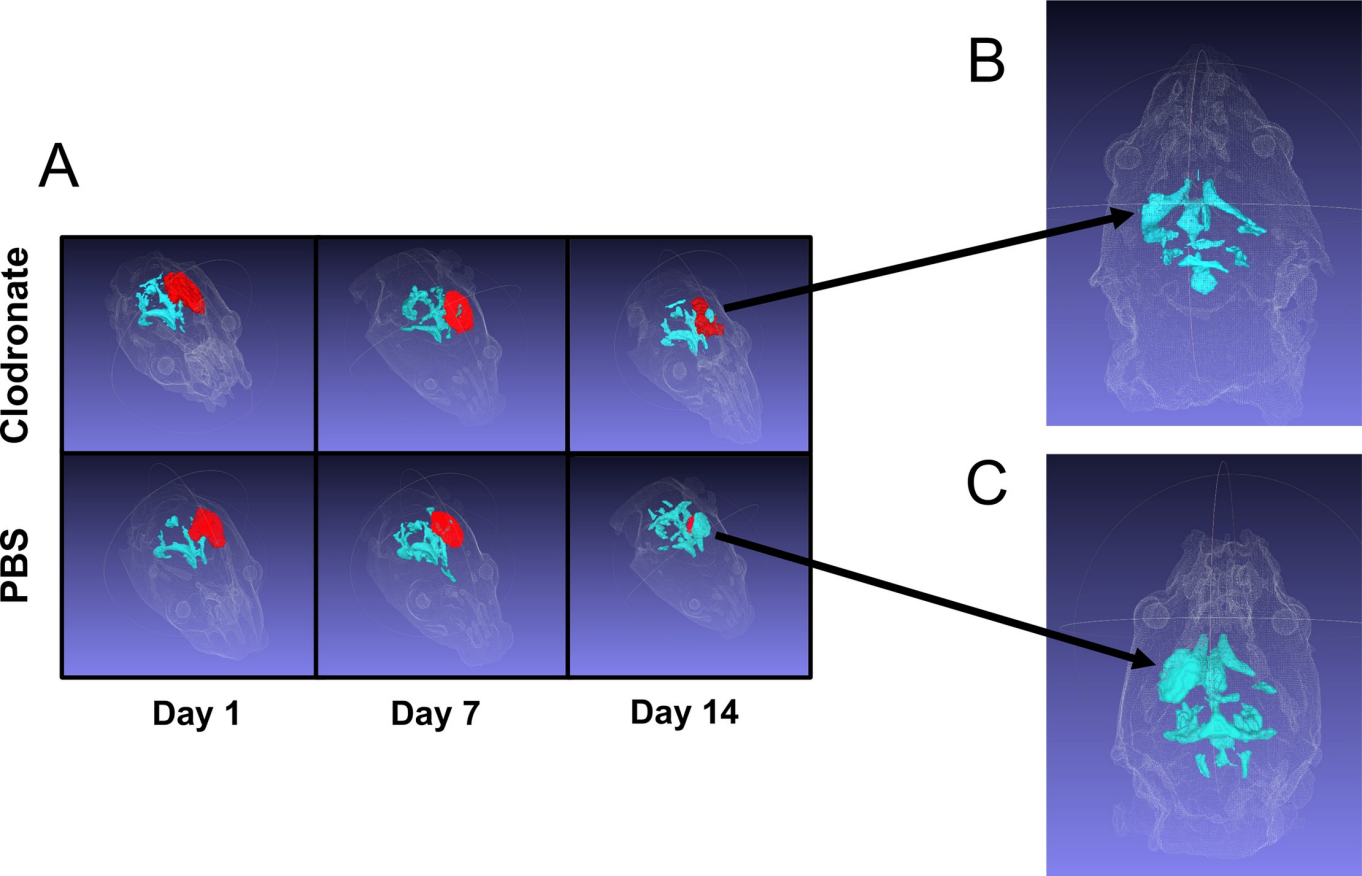
3D renderings of ventricle enlargement in monocyte-depleted vs. sham-depleted mice after traumatic brain injury. **A)** Shown in RED is the extent of traumatically injured tissue at post injury days 1, 7, and 14. Shown in light BLUE is the ventricle size. There is a progressive increase in ventricle size and decrease in injured tissue indicating loss of brain tissue and replacement with cerebrospinal fluid over the course of injury. At 14 days post-TBI the increase in ventricle size between **B)** monocyte-depleted mice (5.3 ± 0.9mm^3^) and **C)** sham-depleted mice (13.2 ± 3.1mm^3^) is markedly attenuated (*p* = 0.02) indicating preservation of the injured neuronal matter.

It is generally accepted that both lesion and ventricle size correlate with poor neurologic outcomes after TBI [28–30]. As discussed above, MRI measurements of tissue classified as “lesion” at 1, 7 and 14 days post TBI did not reveal a strong protective effect from monocyte depletion. Therefore, we aimed to determine whether monocyte depletion attenuated ventricle enlargement after TBI. While there was no difference in ventricle size immediately after injury on post-injury day 1 (reflecting reproducibility of our mechanical TBI induction), a non-statistically significant trend towards decreased ventricle size in monocyte-depleted mice by post-injury day 7 was observed from the MRI data. At 14 days post-injury monocyte depletion had markedly attenuated ventricle enlargement (5.3 ± 0.9mm^3^) as compared to sham-depleted mice (13.2 ± 3.1mm^3^; *p* = 0.02) (**Figs 3D** and **4**). These data suggest that the neurocognitive and locomotor benefits seen after monocyte depletion in our prior work may be secondary to the prevention of posttraumatic hydrocephalus after TBI [15].

### Monocyte depletion improves neural connectivity after TBI

**Fig 5:**
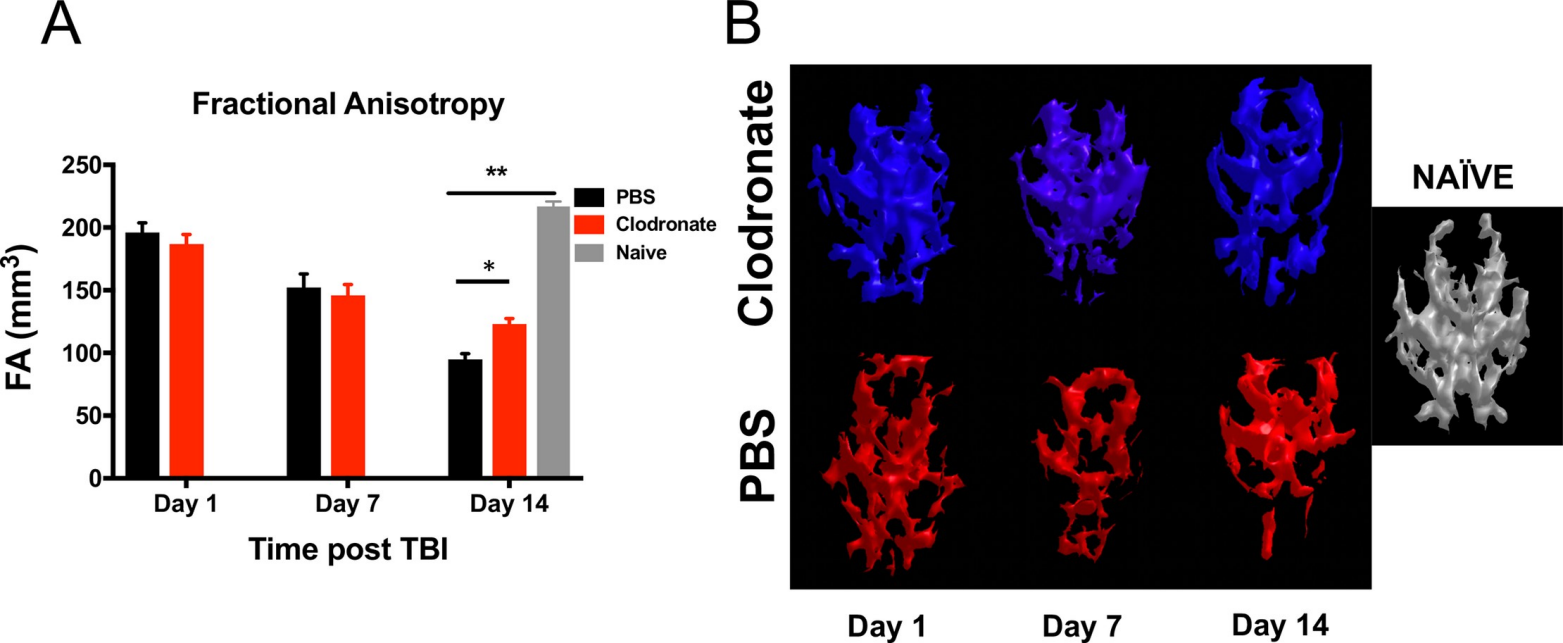
Global fractional anisotropy pattern alterations in monocyte-depleted vs. sham-depleted mice after traumatic brain injury. **A)** At 14 days post—injury the volume of fractional anisotropy above threshold was significantly higher in monocyte-depleted mice (123.0 ± 4.4mm^3^) as compared to sham depleted mice (94.9 ± 4.6mm^3^; * *p* = 0.025), although both were lower compared to Naïve mice (216 ± 3.8mm^3^; ** *p*<0.0001). **B)** Side by side 3D-rendered representative images of fractional anisotropy patterns in monocyte-depleted vs. sham-depleted mice. Noticeable is the progressive loss of pattern as the injury progresses. The volume of FA and a representative 3D-rendered FA pattern from a naïve mouse is provided for comparison.

**Fig 6:**
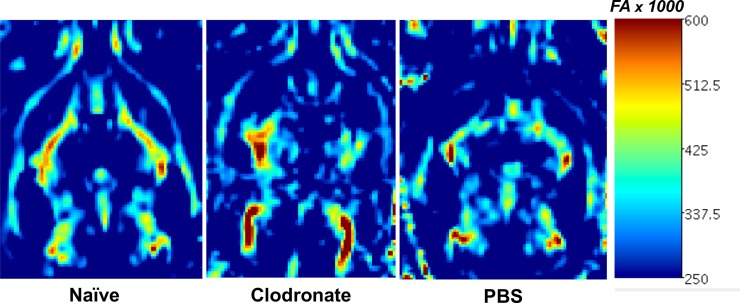
Representative quantitative fractional anisotropy (FA) map 14-days post injury. White arrows showing regions of reduced/altered FA patterns in a sham-depleted mouse as compared to a monocyte-depleted mouse.

Advances in neuroimaging have allowed for in-vivo assessment of traumatic disruptions between neural connections. Given that posttraumatic hydrocephalus leads to cortical thinning and loss of white matter, and that monocyte depletion prevented the development of posttraumatic hydrocephalus, we aimed to determine whether monocyte-depleted mice would show preserved white matter integrity with less disruption of functional networks after TBI. Multi-direction diffusion MRI generates images from which FA maps can be generated. The MR derived FA parameter describes restricted diffusion of water in biological tissue and in the brain has been used to effectively report on fiber density, axonal diameter, and degree of myelination in white matter [31]. Regional reduction of FA values in the brain of mice following TBI can therefore be used as a measure of white matter integrity. To better visualize global FA pattern alterations, we opted to utilize a morphometric 3D representation of FA values for each subject, generated using a threshold method as described in the methods section. This FA image analysis approach allowed us to evaluate white matter changes in the brain-injured mice from each of the two groups. As shown in **Fig 5A**, the tresholded FA volumes (i.e. brain regions with FA values above a certain threshold) was significantly higher in the monocyte-depleted mice (123.0 ± 4.4mm^3^) at 14 days post-injury as compared to sham-depleted mice (94.9 ± 4.6mm^3^; *p* = 0.025) indicating a better preservation of white matter tissue. Also included in the histogram plot in **Fig 5A** is the corresponding average FA value obtained from a cohort of naïve mice (no TBI). Furthermore, both the qualitative 3D morphometric FA maps as well as the quantitative FA map exhibit extensive differences in white matter integrity and patterns between monocyte-depleted and sham-depleted mice and both together show significant differences with respect to the naïve group (**Figs 5B and 6**). The importance of these findings lies in the ability of FA maps to predict deficits in cognitive function based the location of white matter abnormality [32].

## Discussion

While it is clear that the immune response to TBI plays a central role in both the resolution of the initial injury as well as in the propagation of secondary injury, the contribution of infiltrating monocytes has largely been neglected. Although, we have previously published that monocyte depletion reduces cerebral edema, improves memory, and attenuates locomotor dysfunction after TBI, to the best of our knowledge, no immune depletion study to date has deciphered in-vivo anatomic and morphological outcomes during the initiation, progression, and potential resolution of TBI [15]. Magnetic resonance imaging (MRI) is a non-invasive imaging modality that can obtain structural as well as functional information in vivo. MRI utilizes low-energy radio-frequency waves in magnetic fields that allows for significantly higher resolution than ultrasound imaging or computed tomography (CT). Additionally, MRI avoids the ionizing radiation necessary for CT imaging and allows for an unlimited depth of penetration offering an advantage over the optical techniques used in histologic analysis. These advantages make MRI a valuable adjunct to behavioral and histologic assays typically used in preclinical models of disease and injury [33].

We report herein that lesion size, as assessed with contrast-enhanced 3D-MRI, was similar across both monocyte-depleted and sham-depleted mice at 1,7, and 14 days post-TBI (**Fig 3A–3C**). This indicates both a consistent and reproducible lesion across both experimental groups. However, it also suggests that measurement of lesion size by MRI may not be able to reveal the tissue sparing effects of monocyte depletion by direct visualization. This is particularly evident at latter time points when brain tissue loss has occurred and ventricle enlargement initiated. At the same time, monocyte-depleted mice demonstrated relative preservation of ventricle size at 14 days post-injury whereas sham-depleted mice developed significant posttraumatic hydrocephalus (**Figs 3D and 4**). This indicates that monocytes play a significant, yet poorly understood, role in the development of posttraumatic hydrocephalus. The clinical importance of this finding lies in the fact that patients who develop posttraumatic hydrocephalus demonstrate poorer recovery, require more invasive intervention, and have more complications than patients who do not develop posttraumatic hydrocephalus [18, 34, 35]. As we have shown previously, monocyte depletion abrogates neutrophil infiltration into the injured brain, improves learning, memory, motor coordination and skill acquisition [15]. We now show that monocyte depletion likely exerts a tissue-sparing effect which reduces brain tissue atrophy and preserves ventricle volume following TBI thereby attenuating the development of posttraumatic hydrocephalus. In our previous work we detected cellular and neurocognitive/locomotor benefits of monocyte depletion with brain histology and neurocognitive testing. In the current study we are now able to anatomically correlate these findings in-vivo utilizing MRI assay of TBI progression through tracking of ventricular volumes. One observation coming from this study was that longitudinal tracking of the amount of tissue classified as “lesion” did not, as we expected, exhibit a difference between groups. However, given that monocyte depletion significantly attenuated ventricle, we believe that the inability to detect differences between the two groups was primarily associated with the timing of our measurements. In fact, shortly after injury the delineation of hyperintense regions in the brain is relatively straightforward (**Fig 2**). However, at 7 and 14 days post-TBI this becomes more challenging as significant brain loss has already occurred. Current work is focusing on comparing lesion size at higher temporal resolution after TBI (i.e. 1, 2, 3 and 4 days post-TBI) with the expectation that it will be possible to use MRI to tract changes in the tissue microenvironment as neurogenerative processes are taking place.

In addition to in vivo tracking of volumetric and morphometric data, MRI also provides a tool to probe connectivity in the brain of TBI mice [36–38]. Diffusion-weighted MRI probes the diffusion of water within the brain to assess microstructural tissue properties. Because healthy neurons control the direction that water diffuses, diffusion imaging has the ability to map the white matter content in the brain. Differential diffusion within white matter can be used to detect demyelination and axonal degeneration in the form of increased water diffusion through injured myelin structures [39]. This is quantified through fractional anisotropy (FA), and lower FA associates with white matter injury. The FA measurements allow for quantification of different properties of tissue structure and function which can be applied across experimental groups over time [40]. In the current study, our FA data demonstrates that monocyte-depleted mice have significantly lower loss of white-matter compared to sham-depleted mice after TBI (**Fig 5A**). This indicates that monocyte depletion not only attenuates the development of posttraumatic hydrocephalus, but also conserves functional white matter microstructure as shown by preserved quantitative FA volumetrics (**Fig 4**) as well as the qualitative FA maps generated after TBI (**Fig 5B**). These findings correlate well with our prior work demonstrating preserved working memory, better motor coordination, and improved skill acquisition in monocyte-depleted mice [15].

White matter tracts are bundles of axons connecting functionally specific, yet anatomically separated, regions of the brain. When a TBI is sustained, these axons are subjected to shear, compression, and torsion forces. Recent advances in diffusion MRI techniques, such as diffusion tensor imaging (DTI) and high angular resolution diffusion imaging (HARDI), are now allowing for damage to specific white matter bundles to be identified and correlated with neurocognitive functions that are known to associate with those bundles [41]. While the tresholded FA analysis used in the current study is similar to DTI-MRI analysis approaches that can provide fiber tractography visualization, our method has the advantage of a higher sensitivity with respect to DTI techniques. Nonetheless, efforts are underway to compare the two approaches with the aim of exploring specific connectivity patterns using fiber tractography (33).

The fundamental conclusion from this experimental work is that morphological and diffusion MRI allows for the exploration of whole brain tissue alterations (ventricle size and FA patterns) underlying TBI progression in a quantitative and reliable manner. In essence, this set of tools can be used to reliably track and identify macroscopic tissue changes that are associated with sub-cellular and cellular processes involved in neurodegenerative progression. This has the potential to offer better insight into the fundamental evolution of TBI progression allowing for more individualized diagnoses leading to more individualized treatment in the future. Nonetheless, despite the excitement surrounding these new techniques there remains several methodologic obstacles to overcome before cutting edge neuroimaging will be incorporated into patient care [42]. Perhaps the biggest challenge will be for clinicians to incorporate the data generated from these studies into clinical decision making.

## Conclusions

In conclusion, we report that using an MRI assay we did not observe a significant reduction of lesion size following monocyte depletion in TBI mice. However, this result is most likely associated with the fact that MRI’s were conducted at later time-points when brain loss has already occurred and thus direct detection lesioned tissue is challenging. Monocyte depletion clearly attenuates the development of posttraumatic hydrocephalus suggesting that ventricle size is a much more reliable marker of TBI progression than direct assessment of lesion at later time-points. We hypothesize that lesion size as detected by MRI could be more relevant as a biomarker if acquired early on following TBI. In addition, using diffusion MRI and the resultant quantitative FA volumetrics and qualitative FA mapping we show enhanced preservation of functional white matter tracts in monocyte-depleted mice as compared to sham-depleted mice after TBI. These data suggest that peri-injury depletion of monocytes may represent a novel therapeutic strategy for the treatment of TBI with the potential to be translated to human patients.
